# Case Report: 18F-Fluoro-L-Phenylalanine Positron Emission Tomography Findings and Immunoreactivity for L-Type Amino Acid Transporter 1 in a Dog With Meningioma

**DOI:** 10.3389/fvets.2022.899229

**Published:** 2022-07-15

**Authors:** Dohee Lee, Taesik Yun, Sanggu Kim, Yoonhoi Koo, Yeon Chae, Soochong Kim, Dongwoo Chang, Mhan-Pyo Yang, Hakhyun Kim, Byeong-Teck Kang

**Affiliations:** ^1^Laboratory of Veterinary Internal Medicine, College of Veterinary Medicine, Chungbuk National University, Cheongju, South Korea; ^2^Laboratory of Veterinary Pathology and Platelet Signaling, College of Veterinary Medicine, Chungbuk National University, Cheongju, South Korea; ^3^Department of Veterinary Imaging, College of Veterinary Medicine, Chungbuk National University, Cheongju, South Korea

**Keywords:** brain tumor, canine, 18F-FDOPA, L-type amino acid transporter 1, meningioma, positron emission tomography

## Abstract

A 12-year-old intact female Miniature Pinscher dog weighing 5.4 kg presented with a history of seizures. On neurological examination, postural reactions were decreased in the left-sided limbs, and menace responses were bilaterally absent. Magnetic resonance imaging (MRI) of the brain was performed, and a solitary amorphous mass (2.7 × 1.9 × 2.2 cm) was observed on the right side of the frontal lobe. Based on the signalment, clinical signs, and MRI findings, a brain tumor was tentatively diagnosed, and meningioma was suspected. The dog was treated with hydroxyurea, prednisolone, and other antiepileptic drugs. One week after the treatment began, postural reactions returned to normal, and the menace response improved. At 119 days after treatment, 18F-fluoro-L-phenylalanine (18F-FDOPA) positron emission tomography (PET) was performed. Marked 18F-FDOPA uptake was observed in the lesion. The mean and maximal standardized uptake values of the lesion were 2.61 and 3.72, respectively, and the tumor-to-normal tissue ratio was 1.95. At 355 days after the initial treatment, a second MRI scan was performed and the tumor size had increased to 3.5 × 2.8 × 2.9 cm. The dog died 443 days after the initial treatment and was definitively diagnosed with grade 1 meningioma by histopathological examination. Immunohistochemical staining for Ki67 and L-type amino acid transporter 1 was positive and negative for p53, respectively. The labeling index of Ki67 was 2.4%. This is the first case to demonstrate 18F-FDOPA PET findings in a clinical case of a dog histologically diagnosed with a meningioma.

## Introduction

Meningioma is the most common intracranial tumor in dogs, accounting for 45% of primary brain tumors (1, 2). It originates from arachnoid cells of the meninges and causes various clinical signs by compressing parenchymal tissue and secondary effects, such as peritumoral edema and neuroinflammation (2, 3). There are several treatment options for canine meningioma, including surgical resection, radiation, chemotherapy, and palliative treatment with steroids and anticonvulsants (4–6).

In human medicine, positron emission tomography (PET)/computed tomography (CT) using 18F-fluoro-L-phenylalanine (18F-FDOPA) has been used to visualize a variety of neuroendocrine tumors (7, 8). 18F-FDOPA has been proposed as a useful radiotracer for detecting brain tumors owing to its higher sensitivity compared with other radiotracers (9, 10). However, in veterinary medicine, there are few reports of 18F-FDOPA PET findings in brain tumors, except for one report of a dog with a glioma (11). Therefore, this case report is the first to describe 18F-FDOPA PET findings in a clinical case of a dog histologically diagnosed with meningioma. Furthermore, the increased immunoreactivity for L-type amino acid transporter 1 (LAT1) suggests that LAT1 may be a diagnostic target for PET imaging and a therapeutic target in canine meningiomas.

## Case Presentation

A 12-year-old intact female Miniature Pinscher weighing 5.4 kg presented with a history of seizures and aggression. The dog experienced the first seizure 5 months prior, and the seizure progressively frequency increased. Physical examination revealed right-sided exophthalmos. Complete blood cell count and electrolyte analysis were within the normal range. Biochemical analysis results were unremarkable except for increased alkaline phosphatase activity (1,299 IU/L, reference range 29–97 IU/L). The levels of other liver enzymes were normal, and the liver size was normal on survey radiographs. On neurological examination, postural reactions were decreased in the left-sided limbs, and menace responses were absent bilaterally. Based on the clinical signs and findings from neurological examination, the lesion was neuroanatomically localized to the cerebrum, particularly on the right side.

**Figure 1:**
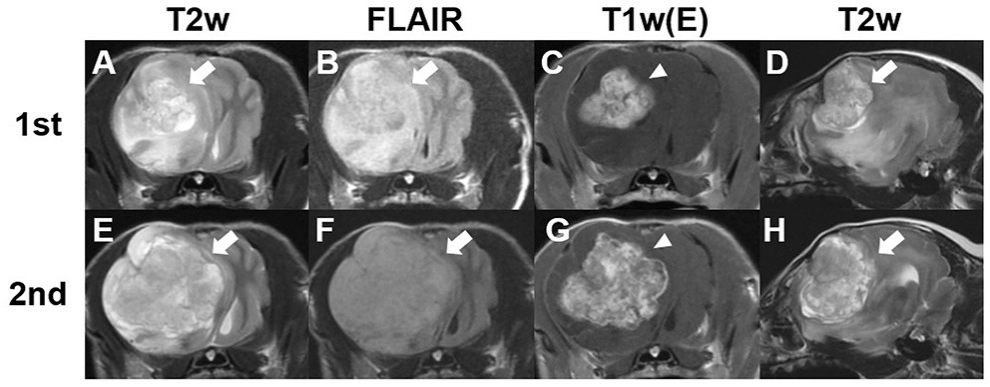
Findings from the first and second magnetic resonance (MRI) imaging of a dog with meningioma. Transverse T2-weighted **(A,E)**, fluid-attenuated inversion recovery **(B,F)**, and post-contrast **(C,G)** images at the optic chiasm level and sagittal T2-weighted images **(D,H)**. The first MRI scan was performed before chemotherapy. A solitary amorphous mass (2.7 × 1.9 × 2.2 cm) can be observed in the right frontal lobe. The tumor lesion (arrows) shows hyperintensity on T2-weighted **(A,D)** and fluid-attenuated inversion recovery **(B)** images. Post-contrast **(C)** image shows uniformly remarkable enhancement (arrowhead). Second MRI was obtained 355 days after initial chemotherapy **(E–H)**. A more severe midline shift compared with the findings of the first MRI scan can be observed and the size of the tumor increased to 3.5 × 2.8 × 2.9 cm.

Magnetic resonance imaging (MRI) (1.5-Tesla unit, Signa Creator, GE Healthcare, Milwaukee, WI, USA) of the brain was performed under general anesthesia with 2% isoflurane (Terrell, Piramal Critical Care, Bethlehem, PA, USA) following the induction of anesthesia with propofol (6 mg/kg; Provive, Myungmoon Pharm. Co., Ltd., Seoul, South Korea). T1-weighted (pre- and post-contrast), T2-weighted, and fluid-attenuated inversion recovery images were obtained in the transverse, sagittal, and dorsal planes. A solitary amorphous mass was observed in the frontal lobe on the right side, which was hypointense to isointense on T1-weighted images and hyperintense on T2-weighted (Figures 1A,D) and fluid-attenuated inversion recovery images (Figure 1B). The mass was well-demarcated, heterogenous, and 2.7 × 1.9 × 2.2 cm in size (width × length × height). It was challenging to distinguish the origin of the mass (intra- or extra- axial) because it looked like it originated from the brain parenchyma but was not completely surrounded by normal brain tissue. The mass was peripherally located, in contact with the meninges, and compressed the surrounding brain parenchyma rather than invading it. Midline shift to the left and perilesional edema were also observed. After administration of 0.1 mmol/kg gadolinium-diethylenetriamine penta-acetic acid [Omniscan™, GE Healthcare (Shanghai), Co., Ltd, China], the mass showed strong contrast-enhancement and meningeal enhancement was identified adjacent to the mass (Figure 1C). Cerebrospinal fluid was not obtained because indentation of the cerebellum was observed on MRI and there was a high probability of increased intracranial pressure caused by a large intracranial mass. Based on the signalment, history, clinical signs, and MRI findings, a brain tumor was tentatively diagnosed, and the differential diagnoses included meningioma, glioma, and choroid plexus tumor.

Surgical removal or radiation was not performed because of the deep location and size of the tumor, as well as financial constraints of the owner. Instead, oral chemotherapy with hydroxyurea, combined with supportive treatment with prednisolone and antiepileptic drugs, was administered. Hydroxyurea was initially selected as chemotherapeutic agent because meningioma and glioma were considered a likely differential and the drug has the advantage of low cost and being relatively well tolerated; imatinib was not used at this time due to the high cost and financial constraint of the dog's owner. The dog was initially treated with hydroxyurea 50 mg/kg q48 h (Hydrin®, Korea United Pharm., Seoul, South Korea), prednisolone 0.5 mg/kg q12 h (Solondo®, Yuhan, Seoul, South Korea), and phenobarbital 3 mg/kg q12 h (Phenobarbital, Hana Pharm CO., Seoul, South Korea). One week after treatment initiation, postural reactions returned to normal, and the menace response improved. At 28 days after treatment initiation, elevated hepatic enzyme levels were noted, and the dose of prednisolone was decreased to 0.5 mg/kg q24 h. At 34 days after the initial therapy, the dog showed cluster seizures and increased hepatic enzyme levels. To control seizures and decrease liver toxicity, the dog was administered levetiracetam 10 mg/kg q8 h (Keppra®, UCB Pharma, Bruxelles, Belgium) and a maintenance dose of potassium bromide (15 mg/kg 12 h; Potassium bromide, Sigma-Aldrich Co., Steinheim, Germany) after 5 days loading dose (60 mg/kg q12 h). The phenobarbital dose was tapered over 3 weeks.

The dog's clinical signs were well controlled, and there were no side effects from chemotherapy with hydroxyurea, prednisolone, or antiepileptic drugs (potassium bromide, levetiracetam). At 119 days after the initiation of treatment, 18F-FDOPA PET was performed under general anesthesia to evaluate the metabolic activity of the lesion and to determine the change in size of the contrast-enhanced lesion. General anesthesia was induced with intravenous administration of propofol (4 mg/kg; Provive, Myungmoon Pharm. Co., Ltd., Seoul, South Korea) and maintained with 2.5% isoflurane (Terrell, Piramal Critical Care, Inc., Bethlehem, PA, USA). 18F-FDOPA (3.5 MBq/kg) was administered intravenously into the saphenous vein (10), followed by 5 mL of 0.9% normal saline flushing of residual 18F-FDOPA. Low-dose CT images (pre- and post-contrast) were obtained prior to the PET scan (Discovery-STE, General Electric Medical Systems, Waukesha, WI, USA). Twenty-minute PET images were acquired 10 min after 18F-FDOPA injection (10). PET images were analyzed using a commercial program (OsiriX MD v10.0; Pixmeo Sarl, Geneva, Switzerland). The regions of interest were drawn manually on the PET/CT fusion images, and the metabolic activity inside the regions of interest was converted to a standardized uptake value (SUV) as follows: SUV = average tissue concentration of 18F-FDOPA (MBq/ml)/injected dose (MBq) per body weight (g). To objectively evaluate metabolic activity, the tumor-to-normal tissue (T/N) ratio was measured by dividing the maximal SUV of the tumor by the maximal SUV of the normal brain tissue.

**Figure 2:**
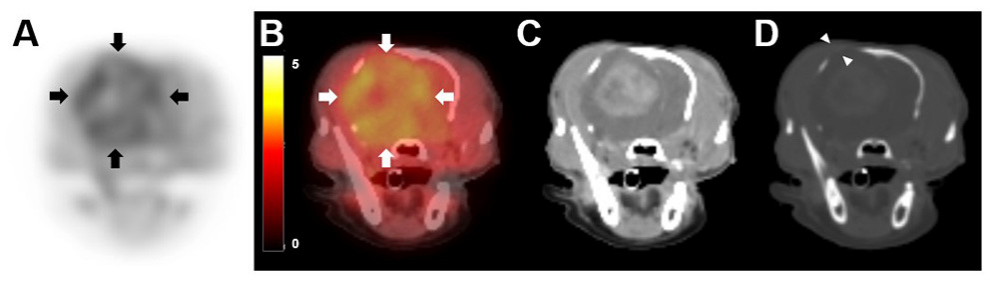
18F-fluorodopa (18F-FDOPA) positron emission tomography (PET)/computed tomography (CT) findings in a dog with meningioma. PET/CT scan was performed 119 days after initial chemotherapy. **(A)** On 18F-FDOPA PET image, increased 18F-FDOPA uptake is shown with a black color, while low uptake is shown with a white color. 18F-FDOPA uptake was remarkable in the lesion (arrows). **(B)** On PET/CT fusion image, increased 18F-FDOPA uptake appeared to be yellow, while low uptake appeared black to red. **(C)** A contrast-enhanced CT image shows an intense enhancement of the mass. **(D)** Bone window CT after contrast medium injection (window width of 2,000 Hounsfield units, window level of 500 Hounsfield units) revealed cranial bone lysis around the tumor (arrowhead).

On visual evaluation of the PET images, there was marked 18F-FDOPA uptake in the tumor and peritumoral lesion (Figure 2A). The mean and maximal SUV of the lesion were 2.61 and 3.72, respectively; the tumor region showed the mean SUV 2.57 and maximal SUV 3.72, and peritumoral region showed the mean SUV 2.66 and maximal SUV 3.69 (Figure 2B). The T/N ratio was 1.95. On post-contrast CT images (Figure 2C), the size of the contrast-enhanced lesion was 2.9 × 2.2 × 2.4 cm (width × length × height), and it was smaller than the hypermetabolic 18F-FDOPA lesion (3.5 × 2.2 × 3.3 cm; width × length × height), with an SUV of >1.91, corresponding to maximal SUV of the normal brain area of the dog. The longest diameter of the mass on the post-contrast CT scan increased to 7.4% compared with that on the first MRI scan. CT imaging in a bone window (window width, 2,000 Hounsfield units; window level, 500 Hounsfield units) revealed cranial bone lysis around the tumor (Figure 2D).

On day 238 after initial treatment, neurological examination showed decreased postural reaction in the left forelimb, which gradually deteriorated. On day 328 after therapy commencement, postural reactions of the left forelimb were absent, and the left hindlimb showed decreased postural reaction. In addition, worsening seizures were observed. Based on deterioration of clinical signs and neurological examination, prednisolone was increased to 0.5 mg/kg q12 h and levetiracetam was increased to 20 mg/kg q8 h. While the seizures were well controlled, there was no improvement of neurological abnormality, and the dose of prednisolone was reduced to 0.5 mg/kg q24 h due to poor response and increased liver enzymes.

On day 355 after the initial treatment, a second MRI scan was performed to evaluate tumor size (Figures 1E–H). The second MRI showed severe left side midline shift compared with previous imaging, and the tumor size had increased to 3.5 × 2.8 × 2.9 cm (width × length × height), corresponding to a 29.6% increase in the longest diameter compared with the first MRI scan and an 20.7% increase in the longest diameter compared with the post-contrast CT scan. The perilesional edema was more extensive than before. Owing to the increase in tumor size despite the administration of hydroxyurea, imatinib (8 mg/kg, q24 h; Glima®, Boryung Pharmaceutical Co., Ltd., Seoul, South Korea) was added to the previous treatment. The dog died from acute kidney injury and severe acute pancreatitis 443 days after the initial treatment.

**Figure 3:**
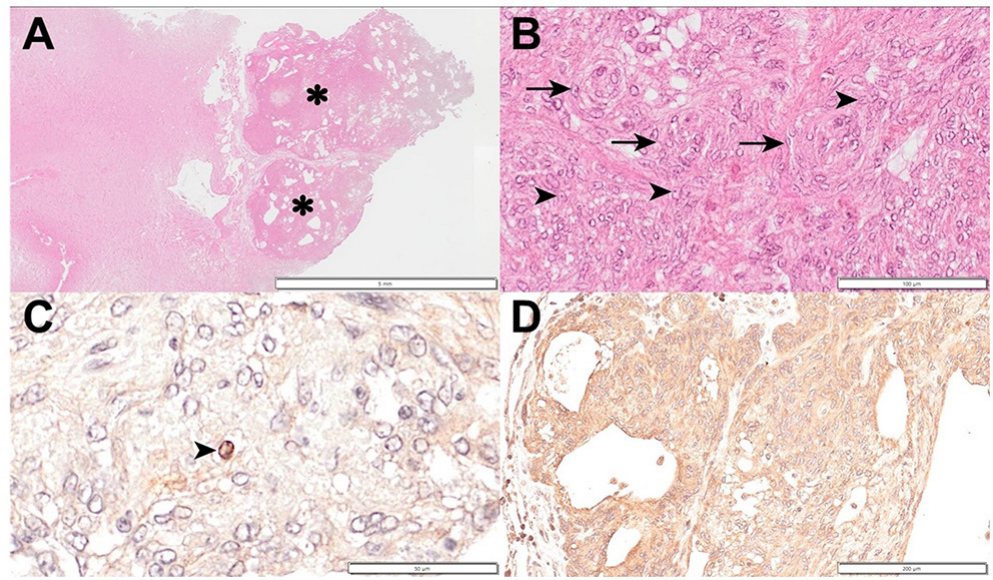
Histopathology and immunohistochemical evaluation of a meningioma in a dog. **(A)** Normal brain tissue was compressed by an encapsulated neoplastic mass (asterisk). Hematoxylin and eosin; ×6 magnification; bar = 5 mm. **(B)** Syncytial growth of round to oval meningothelial cells consisting solid sheet and meningeal whorls (arrow). Nuclei contain a clear space with prominent nucleoli (arrowhead). Hematoxylin and eosin; ×200 magnification; bar = 100 μm. **(C)** A Ki67 positive cell (arrowhead) can be observed. Hematoxylin counterstain; ×400 magnification; bar = 50 μm. **(D)** L-type amino acid transporter 1 stain showing strongly diffuse positive staining. Hematoxylin counterstain; ×100 magnification; bar = 200 μm.

The owner did not agree to a necropsy but agreed to sampling the tissue of the brain lesion through the lysed cranial bone. Histopathological examination revealed that the normal brain tissue was compressed by an encapsulated neoplastic mass (Figure 3A). The syncytial growth of round to oval meningothelial cells consisted of a solid sheet and meningeal whorls. The nuclei contained clear spaces with prominent nucleoli (Figure 3B). A few mitoses (<4/10 high-power fields) were also observed. Based on these histological findings and the World Health Organization (WHO) classification, the lesion was definitively diagnosed as grade 1 meningioma (12). Immunohistochemical (IHC) staining for Ki67, a proliferation index, p53, an index of cell cycle regulation, and LAT1 was performed. Neoplastic cells were positive for Ki-67 (Figure 3C) and negative for p53. IHC labeling for Ki67 revealed strong nuclear labeling of 24 cells per 1,000; therefore, the labeling index of Ki67 and p53 was 2.4 and 0%, respectively, which further confirmed that the tumor was grade 1 meningioma (13–16). LAT1 IHC staining showed strong, diffuse positive staining (Figure 3D).

## Discussion

In the present case, the dog was suspected to have a brain tumor and survived for 443 days on chemotherapy with hydroxyurea and prednisolone, with the later addition of imatinib. Intracranial meningioma was definitively diagnosed based on histological examination. This is the first case report to demonstrate 18F-FDOPA PET findings in a dog with a naturally occurring meningioma.

PET/CT is useful for differentiating benign and malignant lesions (17), and 18F-FDG, a glucose analog, is the most widely used PET tracer. However, its diagnostic utility for brain tumors is limited because the high physiological metabolic rate of glucose in normal brain tissue leads to a high uptake of 18F-FDG in the cerebral parenchyma, which obscures the visualization of malignancy (18). 18F-FDOPA, a new amino acid analog, has been suggested as an alternative to 18F-FDG for the evaluation of brain tumors (9, 10). A previous study reported that the sensitivity of 18F-FDOPA (96%) for the detection of brain tumors was significantly higher than that of 18F-FDG (61%), particularly for low-grade tumors (10). In addition, the uptake of 18F-FDOPA in tumors was more rapid than 18F-FDG uptake. A previous study of human brain tumors, 18F-FDG showed peak activity approximately 60 min after intravenous injection, while 18F-FDOPA showed peak activity 15 min after injection (10). In that study, 18F-FDOPA 3.5 Mbq/kg was injected intravenously, and tumor activity peaked between 10 (98%) and 30 (93%) min after the injection and declined after that (10). Based on that results, 18F-FDOPA dose 3.5 Mbq/kg was chosen, and images between 10 and 30 min after 18F-FDOPA administration were obtained. 18F-FDOPA may be clinically more useful for imaging canine brain tumors than 18F-FDG, as most small animals need general anesthesia for PET imaging and brain tumors usually occur in elderly dogs with a high risk of general anesthesia.

L-DOPA is a precursor of the neurotransmitter dopamine, and 18F-FDOPA, which is radiolabeled L-DOPA with the position emission isotope 18F, has the same metabolism as L-DOPA. These amino acids are taken up by LAT1, an important transporter of various amino acids into cells (19). LAT1 is expressed in endothelial cells of the blood–brain barrier (20), but its expression is low in normal brain tissue (21). In comparison, it is overexpressed in tumor cell lines that require many amino acids for proliferation (22), and its association with tumor growth has been proven (23). In a previous human study of brain tumors, upregulated LAT1 was associated with significant 18F-FDOPA uptake (24). Although there are a few cases of 18F-FDOPA uptake in human meningiomas (25, 26), immunoreactivity for LAT1 has been previously reported in human meningiomas (27). In addition, a dog with glioma showed 18F-FDOPA uptake and LAT1 expression (11). Therefore, whether the tumor was meningioma or glioma, we predicted that the tumor's metabolic function could be evaluated with 18F-FDOPA PET, in the present dog. The present case showed increased 18F-FDOPA metabolism and revealed strongly diffuse immunoreactivity for LAT1. In veterinary medicine, immunoreactivity for LAT1 in meningiomas was evaluated in only two dogs and two brain samples showed negative or weakly positive reactions for LAT1, previously (28). This is the first case in canine meningioma to show marked immunoreactivity for LAT1, 18F-FDOPA uptake, and suggests that LAT1 may be a molecular target for diagnostic PET imaging and a therapeutic target for canine meningioma.

Due to the lack of 18F-FDOPA PET data in small animal and limited 18F-FDOPA PET data in human meningioma, 18F-FDOPA uptake in the present case was compared with the study of 18F-FDOPA PET imaging in human glioma. In a previous study on humans, mean SUV of normal brain tissue was 1.69 ± 0.11 and 1.21 ± 0.07 in gray and white matter, respectively (10). In the present case, the mean SUV of the lesion was 2.61, which was higher than that of normal human brain tissue previously reported (10). The same study reported that a T/N ratio > 1.3 demonstrated a high sensitivity (96%) and specificity (86%) for the identification of brain tumors in human medicine (10). The T/N ratio of the present dog was 1.95, consistent with the value in human brain tumors, and meningioma was confirmed by histopathologic examination. In this study, there was no marked difference in 18F-FDOPA uptake between high- and low-grade gliomas, but most of the cases included had performed PET examinations after recurrence (10). In another study on human patients with newly diagnosed gliomas, tumor uptake on 18F-FDOPA was considerably higher in high-grade gliomas than in low-grade gliomas (maximal SUV 4.22 ± 1.30 in high-grade and 2.67 ± 1.18 in low-grade gliomas) (29). A cutoff of 2.72 for maximal SUV of the tumor differentiated low-grade from high-grade gliomas with 85% sensitivity and 89% specificity (29). In a previous case of a dog with low-grade glioma, 18F-FDOPA PET showed the maximal SUV of the tumor as 2.29 and a T/N ratio of 2.22 (11), which were similar to the results of a previous human study (29). In the present case, the meningioma was grade 1 according to the WHO classification (12) and the maximal SUV of the tumor and T/N ratio was 3.72 and 1.95, respectively. Further studies are needed to determine whether 18F-DOPA PET uptake can distinguish histologic grade of canine meningioma and this case may provide fundamental information for it.

PET has several advantages over conventional imaging modalities such as CT or MRI, showing only anatomic lesion, in that it provides functional information of tumors (30). 18F-FDOPA PET allowed for a more accurate delineation of the brain tumor margins (31). In addition, metabolic tumor volume obtained from 18F-FDOPA images provides useful information to predict tumor recurrence or progression, evaluate treatment response, and for surgical planning in human brain tumors (31–33). In the present case, the region showing increased 18F-FDOPA uptake in PET images was wider than the contrast-enhanced region in CT images. Conversely, a functional lesion was wider than the gross lesion on PET/CT images, and after around 6 months, a second MRI presented tumor growth and expanded contrast-enhanced area. One case similar to the present case was reported in human glioma (34). In a case, the lesion showed abnormal 18F-FDOPA activity in a broad area, and it includes the narrow region of contrast enhancement and surrounding non-contrast enhancing parenchyma (34). On MRI after 3 months, contrast enhancement was extended to a site that previously exhibited abnormal 18F-FDOPA activity without contrast enhancement (34). These two cases suggest that 18F-FDOPA metabolism may precede contrast enhancement, which may be associated with the progression of the tumor, as previously reported (33).

Grade 1 meningioma is less invasive (12), whereas the mean 18F-FDOPA uptake in the peritumoral region was higher than in the tumor region. The causes of this contradiction are presumed to be as follows. First, there is a possibility that the functional metabolism of the peritumoral region was increased before progression to tumor, destruction of blood-brain barrier, or increase in vascularity. Second, it may be the effect of inflammation around the tumor. In human medicine, two patients suspected of low-grade brain tumors that showed abnormal 18F-FDOPA uptake confirmed as brain inflammation based on histopathological examination (35). The possibility of the contribution of inflammation to 18F-FDOPA should be considered because inflammation may also contribute to the 18F-FDOPA uptake (35), and findings of peritumoral inflammation were shown in the first and second MRI scans.

To the best of our knowledge, there are no previous reports of canine meningiomas identified by 18F-FDOPA PET, and there has been only one report of a dog with glioma presented by 18F-FDOPA PET in veterinary medicine (11). Therefore, this case is the first to demonstrate 18F-FDOPA PET findings in a clinical case of a dog histologically diagnosed with a meningioma. Furthermore, the tumor lesions revealed increased immunoreactivity for LAT1, suggesting that LAT1 may be a diagnostic target for PET imaging and a therapeutic target in canine meningiomas.

## Data Availability Statement

The original contributions presented in the study are included in the article/supplementary material, further inquiries can be directed to the corresponding author.

## Ethics Statement

Ethical review and approval was not required for the animal study because the study was a case report. Written informed consent was obtained from the owners for the participation of their dog in this study.

## Author Contributions

DL, TY, YK, YC, DC, M-PY, HK, and B-TK analyzed and interpreted the patient data. DL was the main contributor in writing the manuscript. DL, TY, YK, YC, HK, and B-TK evaluated 18F-FDOPA PET/CT images and contributed to image descriptions and discussion in this manuscript. SaK and SoK performed histopathological evaluation. All authors have approved this manuscript.

## Funding

This work was supported by Korea Institute of Planning and Evaluation for Technology in Food, Agriculture, Forestry (IPET) through Companion Animal Life Cycle Industry Technology Development Program, funded by Ministry of Agriculture, Food and Rural Affairs (MAFRA) (322095-04).

## Conflict of Interest

The authors declare that the research was conducted in the absence of any commercial or financial relationships that could be construed as a potential conflict of interest.

## Publisher's Note

All claims expressed in this article are solely those of the authors and do not necessarily represent those of their affiliated organizations, or those of the publisher, the editors and the reviewers. Any product that may be evaluated in this article, or claim that may be made by its manufacturer, is not guaranteed or endorsed by the publisher.

## Abbreviations

CT: computed tomography
18F-FDG: 18F-fluorodeoxyglucose
18F-FDOPA: 18F-fluoro-l-phenylalanine
IHC: immunohistochemical
LAT1: L-type amino acid transporter 1
MRI: magnetic resonance imaging
PET: positron emission tomography
SUV: standardized uptake value
T/N: tumor-to-normal tissue.
